# Supplementary figures and images for: Does individual advocacy work?: A research and evaluation protocol for a youth anti-sex trafficking program
Source: PLoS One. 2022 Jun 29;17(6):e0270103. doi: 10.1371/journal.pone.0270103 (PMC9242468; doi:10.1371/journal.pone.0270103)

**
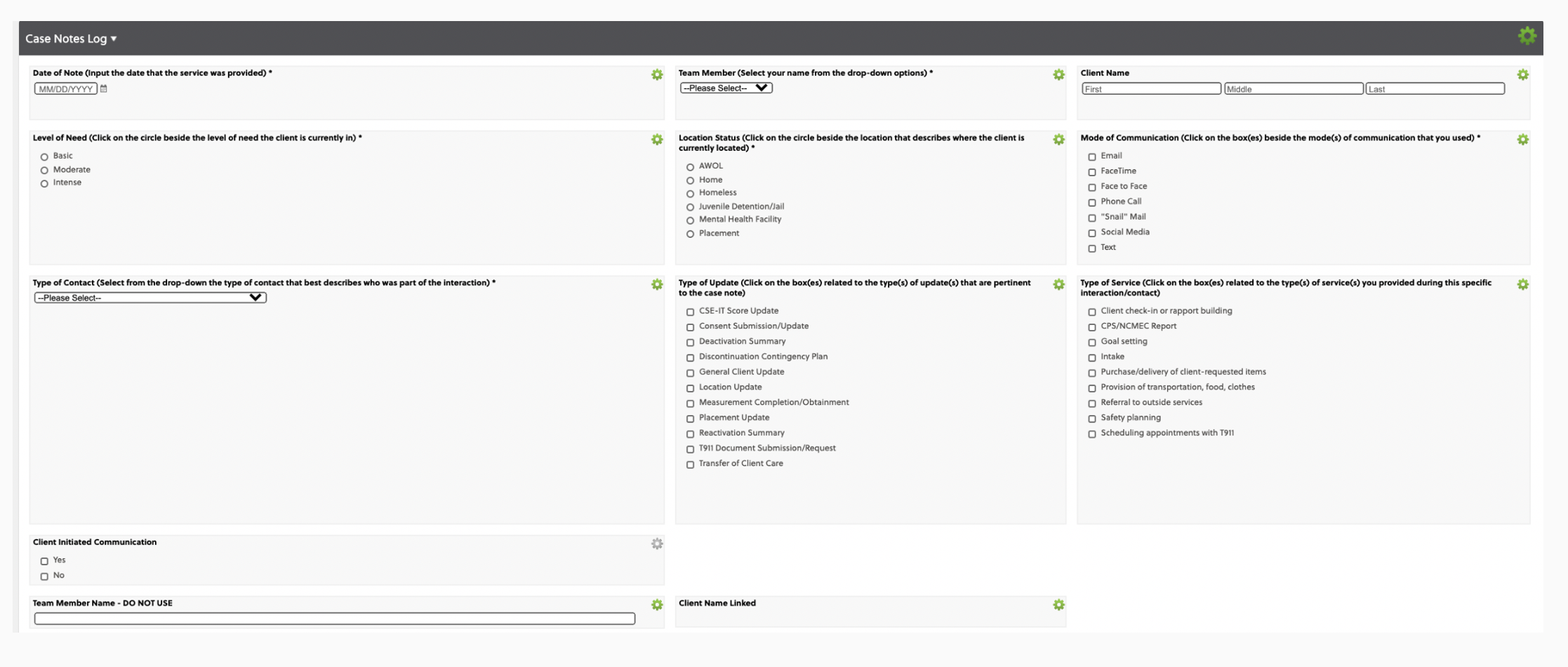
**

Supplement: S1 Fig — Screenshot of the case note interface form in Apricot. (DOCX) [file pone.0270103.s001.docx]
