# Supplementary material for: Does individual advocacy work?: A research and evaluation protocol for a youth anti-sex trafficking program
Source: PLoS One. 2022 Jun 29;17(6):e0270103. doi: 10.1371/journal.pone.0270103 (PMC9242468; doi:10.1371/journal.pone.0270103)
Supplement: S2 Fig — Form for goal development with clients. (DOCX) [file pone.0270103.s002.docx]

| Goal | Type of Goal | | Actions to Take | Date Goal was Set | Date Goal was Achieved |
| --- | --- | --- | --- | --- | --- |
|  |  |  |  | ___ /___ /___ | ___ /___ /___ |
|  | Safety and Security Goal |  |  |  |  |
|  | Physiological Needs Goal |  |  |  |  |
|  | Personal Goal |  |  |  |  |
|  |  |  |  |  |  |
