# Supplementary material for: Does individual advocacy work?: A research and evaluation protocol for a youth anti-sex trafficking program
Source: PLoS One. 2022 Jun 29;17(6):e0270103. doi: 10.1371/journal.pone.0270103 (PMC9242468; doi:10.1371/journal.pone.0270103)
Supplement: S1 File — URICA measure in English and Spanish. (DOCX) [file pone.0270103.s003.docx]

University of Rhode Island Change Assessment Scale – The Game

Please indicate the extent to which you tend to agree or disagree with each statement inserting the behavior you chose above. In each case, make your choice in terms of how you feel right now, not what you have felt in the past or would like to feel.

**There are five possible responses to each of the items in the questionnaire:**

1 = Strongly Disagree

2 = Disagree

3 = Undecided

4 = Agree

5 = Strongly Agree

**Instructions**: Circle the number that best describes how much you agree or disagree with each statement.

|  | Strongly disagree | Disagree | Neither agree nor disagree | Agree | Strongly agree |
| --- | --- | --- | --- | --- | --- |
| It doesn’t make sense for me to consider quitting the game. | 1 | 2 | 3 | 4 | 5 |
| I’ve been thinking I might want to quit the game. | 1 | 2 | 3 | 4 | 5 |
| At times me being in the game causes problems and I’m determined to change. | 1 | 2 | 3 | 4 | 5 |
| I thought I resolved the reasons why I am in the game, but I’m still struggling with those reasons. | 1 | 2 | 3 | 4 | 5 |
| Trying to quit the game is pretty much a waste of time for me. | 1 | 2 | 3 | 4 | 5 |
| I may have faults but there’s nothing I really need to change about me being in the game. | 1 | 2 | 3 | 4 | 5 |
| I thought once I stopped the game. I’d be free of it, but sometimes I’m still struggling with it. | 1 | 2 | 3 | 4 | 5 |
| I may have a problem with being in the game that I should work on. | 1 | 2 | 3 | 4 | 5 |
| I’m really working hard to quit the game. | 1 | 2 | 3 | 4 | 5 |
| I hope that someone will have good advice about quitting the game. | 1 | 2 | 3 | 4 | 5 |
| Anyone can talk about quitting the game, but I’m actually going to do something about it. | 1 | 2 | 3 | 4 | 5 |
| After all I’ve done to quit the game every now and then it comes back to haunt me. | 1 | 2 | 3 | 4 | 5 |

Escala de Evaluación de Cambios de la Universidad de Rhode Island – El Juego

Las siguientes afirmaciones describen cómo se puede sentir una persona al iniciar los servicios de apoyo. Indique en qué medida tiende a estar de acuerdo o en desacuerdo con cada afirmación en función de cómo se siente en este momento, no de lo que sintió en el pasado o le gustaría sentir.

**Hay cinco posibles respuestas a cada una de las declaraciones del cuestionario:**

1 = Totalmente en desacuerdo

2 = En desacuerdo

3 = Ni de acuerdo, ni en desacuerdo

4 = De acuerdo

5 = Totalmente de acuerdo

**Instrucciones**: Marque con un círculo el número que mejor describa su grado de acuerdo o desacuerdo con cada afirmación.

|  | Totalmente  en  desacuerdo | En  desacuerdo | Ni de acuerdo,  ni en desacuerdo | De acuerdo | Totalmente de acuerdo |
| --- | --- | --- | --- | --- | --- |
| No tiene sentido que considere dejar **el juego.** | 1 | 2 | 3 | 4 | 5 |
| Pienso que quizás quiera dejar **el juego.** | 1 | 2 | 3 | 4 | 5 |
| A veces, estar en **el juego** causa problemas y estoy decidido/a a cambiar. | 1 | 2 | 3 | 4 | 5 |
| Pensé que había resuelto los motivos por los que estoy en **el juego**, pero todavía sigo luchando contra esos motivos. | 1 | 2 | 3 | 4 | 5 |
| Intentar dejar **el juego** es una pérdida de tiempo para mí. | 1 | 2 | 3 | 4 | 5 |
| Puede que tenga defectos, pero no hay nada que realmente necesite cambiar para participar **del juego.** | 1 | 2 | 3 | 4 | 5 |
| Pensé que una vez que dejara **el juego** sería libre, pero a veces todavía me encuentro luchando contra él. | 1 | 2 | 3 | 4 | 5 |
| Es posible que tenga un problema sobre el cual debo trabajar relacionado con estar **en el juego.** | 1 | 2 | 3 | 4 | 5 |
| Realmente estoy esforzándome para dejar **el juego.** | 1 | 2 | 3 | 4 | 5 |
| Espero que alguien tenga buenos consejos sobre cómo dejar **el juego.** | 1 | 2 | 3 | 4 | 5 |
| Es fácil hablar sobre dejar **el juego**, pero yo realmente voy a hacer algo al respecto. | 1 | 2 | 3 | 4 | 5 |
| Después de todo lo que he hecho para dejar **el juego,** de vez en cuando vuelve a perseguirme. | 1 | 2 | 3 | 4 | 5 |

University of Rhode Island Change Assessment Scale (Risky Behaviors)

Please select a risky behavior from the list below that you feel you would be most applicable to your choices right now.

- Running away
- Substance Abuse
- Connecting online with strangers
- Engaging in risky sexual behavior

Please indicate the extent to which you tend to agree or disagree with each statement inserting the behavior you chose above. In each case, make your choice in terms of how you feel right now, not what you have felt in the past or would like to feel.

**There are five possible responses to each of the items in the questionnaire:**

1 = Strongly Disagree

2 = Disagree

3 = Undecided

4 = Agree

5 = Strongly Agree

**Instructions**: Circle the number that best describes how much you agree or disagree with each statement.

|  | Strongly disagree | Disagree | Neither agree nor disagree | Agree | Strongly agree |
| --- | --- | --- | --- | --- | --- |
| It doesn’t make sense for me to consider quitting **____________.** | 1 | 2 | 3 | 4 | 5 |
| I’ve been thinking I might want to quit _________. | 1 | 2 | 3 | 4 | 5 |
| At times me being in **__________** causes problems and I’m determined to change. | 1 | 2 | 3 | 4 | 5 |
| Anyone can talk about quitting **__________**, but I’m actually going to do something about it. | 1 | 2 | 3 | 4 | 5 |
| After all I’ve done to quit **_______**, every now and then it comes back to haunt me. | 1 | 2 | 3 | 4 | 5 |
| I thought I resolved the reasons why I am in **______**, but I’m still struggling with those reasons. | 1 | 2 | 3 | 4 | 5 |
| Trying to quit **_______** is pretty much a waste of time for me. | 1 | 2 | 3 | 4 | 5 |
| I may have faults but there’s nothing I really need to change about me being in **__________**. | 1 | 2 | 3 | 4 | 5 |
| I thought once I stopped **______** I’d be free of it, but sometimes I’m still struggling with it. | 1 | 2 | 3 | 4 | 5 |
| I may have a problem with being in **__________** that I should work on. | 1 | 2 | 3 | 4 | 5 |
| I’m really working hard to quit **______**. | 1 | 2 | 3 | 4 | 5 |
| I hope that someone will have good advice about quitting **_______.** | 1 | 2 | 3 | 4 | 5 |
| Anyone can talk about quitting **________**, but I’m actually going to do something about it. | 1 | 2 | 3 | 4 | 5 |
| After all I’ve done to quit **_________**, every now and then it comes back to haunt me. | 1 | 2 | 3 | 4 | 5 |

Escala de Evaluación de Cambios de la Universidad de Rhode Island – Comportamiento de riesgo

Las siguientes afirmaciones describen cómo se puede sentir una persona al iniciar los servicios de apoyo. Indique en qué medida tiende a estar de acuerdo o en desacuerdo con cada afirmación en función de cómo se siente en este momento, no de lo que sintió en el pasado o le gustaría sentir.

**Hay cinco posibles respuestas a cada una de las declaraciones del cuestionario:**

1 = Totalmente en desacuerdo

2 = En desacuerdo

3 = Ni de acuerdo, ni en desacuerdo

4 = De acuerdo

5 = Totalmente de acuerdo

**Instrucciones**: Marque con un círculo el número que mejor describa su grado de acuerdo o desacuerdo con cada afirmación.

|  | Totalmente  en  desacuerdo | En  desacuerdo | Ni de acuerdo,  ni en desacuerdo | De acuerdo | Totalmente de acuerdo |
| --- | --- | --- | --- | --- | --- |
| No tiene sentido que yo considere dejar _______**.** | 1 | 2 | 3 | 4 | 5 |
| He estado pensando que quizás quiera dejar _________**.** | 1 | 2 | 3 | 4 | 5 |
| A veces, estar en **__________** causa problemas y estoy decidido/a a cambiar. | 1 | 2 | 3 | 4 | 5 |
| Pensé que había resuelto los motivos por los que estoy en **________**, pero todavía sigo luchando contra esos motivos. | 1 | 2 | 3 | 4 | 5 |
| Intentar dejar _______ es una pérdida de tiempo para mí. | 1 | 2 | 3 | 4 | 5 |
| Puede que tenga defectos, pero no hay nada que realmente necesite cambiar para participar del ______**.** | 1 | 2 | 3 | 4 | 5 |
| Pensé que una vez que dejara ______ sería libre, pero a veces todavía me encuentro luchando contra él. | 1 | 2 | 3 | 4 | 5 |
| Es posible que tenga un problema en el cual debo trabajar relacionado con estar _________**.** | 1 | 2 | 3 | 4 | 5 |
| Realmente estoy esforzándome para dejar ______**.** | 1 | 2 | 3 | 4 | 5 |
| Espero que alguien tenga buenos consejos sobre cómo dejar _______**.** | 1 | 2 | 3 | 4 | 5 |
| Es fácil hablar sobre dejar _______, pero yo realmente voy a hacer algo al respecto. | 1 | 2 | 3 | 4 | 5 |
| Después de todo lo que he hecho para dejar _____**,** de vez en cuando vuelve a perseguirme. | 1 | 2 | 3 | 4 | 5 |
