# Supplementary material for: Does individual advocacy work?: A research and evaluation protocol for a youth anti-sex trafficking program
Source: PLoS One. 2022 Jun 29;17(6):e0270103. doi: 10.1371/journal.pone.0270103 (PMC9242468; doi:10.1371/journal.pone.0270103)
Supplement: S2 File — MSPSS measure in English and Spanish. (DOCX) [file pone.0270103.s004.docx]

Multidimensional Scale of Perceived Social Support

**Instructions**: Read each statement carefully. Indicate how you feel about each statement.

Circle 1 for **very strongly disagree**

Circle 2 for **strongly disagree**

Circle 3 for **mildly disagree**

Circle 4 for **neutral**

Circle 5 for **mildly agree**

Circle 6 for **strongly agree**

Circle 7 for **very strongly agree**

|  | Very Strongly Disagree | Strongly Disagree | Mildly Disagree | Neutral | Mildly Agree | Strongly Agree | Very Strongly Agree |
| --- | --- | --- | --- | --- | --- | --- | --- |
| There is a special person who is around when I am in need. | 1 | 2 | 3 | 4 | 5 | 6 | 7 |
| There is a special person with whom I can share joys and sorrows. | 1 | 2 | 3 | 4 | 5 | 6 | 7 |
| My family really tries to help me. | 1 | 2 | 3 | 4 | 5 | 6 | 7 |
| I get the emotional help & support I need from my family. | 1 | 2 | 3 | 4 | 5 | 6 | 7 |
| I have a special person who is a real source of comfort to me. | 1 | 2 | 3 | 4 | 5 | 6 | 7 |
| My friends really try to help me. | 1 | 2 | 3 | 4 | 5 | 6 | 7 |
| I can count on my friends when things go wrong. | 1 | 2 | 3 | 4 | 5 | 6 | 7 |
| I can talk about my problems with my family. | 1 | 2 | 3 | 4 | 5 | 6 | 7 |
| I have friends with whom I can share my joys and sorrows. | 1 | 2 | 3 | 4 | 5 | 6 | 7 |
| There is a special person in my life who cares about my feelings. | 1 | 2 | 3 | 4 | 5 | 6 | 7 |
| My family is willing to help me make decisions. | 1 | 2 | 3 | 4 | 5 | 6 | 7 |
| I can talk about my problems with my friends. | 1 | 2 | 3 | 4 | 5 | 6 | 7 |

Escala Multidimensional de Apoyo Social Percibido (Social Support)

**Instrucciones:** Lea atentamente cada afirmación. Indique cómo se siente acerca de cada una.

Seleccione 1 para **totalmente en desacuerdo**

Seleccione 2 para **muy en desacuerdo**

Seleccione 3 para **levemente en desacuerdo**

Seleccione 4 para **neutral**

Seleccione 5 para **levemente de acuerdo**

Seleccione 6 para **muy de acuerdo**

Seleccione 7 para **totalmente de acuerdo**

|  | Totalmente en desacuerdo | Muy en desacuerdo | Levemente en desacuerdo | Neutral | Levemente de acuerdo | Muy de acuerdo | Totalmente de acuerdo |
| --- | --- | --- | --- | --- | --- | --- | --- |
| Tengo una persona especial que está cerca cuando la necesito. | 1 | 2 | 3 | 4 | 5 | 6 | 7 |
| Tengo una persona especial con la que puedo compartir mis alegrías y tristezas. | 1 | 2 | 3 | 4 | 5 | 6 | 7 |
| Mi familia realmente intenta ayudarme. | 1 | 2 | 3 | 4 | 5 | 6 | 7 |
| Recibo la ayuda y el apoyo emocional que necesito de mi familia. | 1 | 2 | 3 | 4 | 5 | 6 | 7 |
| Tengo una persona especial que es una verdadera fuente de consuelo para mí. | 1 | 2 | 3 | 4 | 5 | 6 | 7 |
| Mis amigos realmente intentan ayudarme. | 1 | 2 | 3 | 4 | 5 | 6 | 7 |
| Puedo contar con mis amigos cuando las cosas van mal. | 1 | 2 | 3 | 4 | 5 | 6 | 7 |
| Puedo hablar acerca de mis problemas con mi familia. | 1 | 2 | 3 | 4 | 5 | 6 | 7 |
| Tengo amigos con los que puedo compartir mis alegrías y tristezas. | 1 | 2 | 3 | 4 | 5 | 6 | 7 |
| Tengo una persona especial en mi vida que se preocupa por mis sentimientos. | 1 | 2 | 3 | 4 | 5 | 6 | 7 |
| Mi familia está dispuesta a ayudarme a tomar decisiones. | 1 | 2 | 3 | 4 | 5 | 6 | 7 |
| Puedo hablar acerca de mis problemas con mis amigos. | 1 | 2 | 3 | 4 | 5 | 6 | 7 |
