# Supplementary material for: Does individual advocacy work?: A research and evaluation protocol for a youth anti-sex trafficking program
Source: PLoS One. 2022 Jun 29;17(6):e0270103. doi: 10.1371/journal.pone.0270103 (PMC9242468; doi:10.1371/journal.pone.0270103)
Supplement: S3 File — Coping self-efficacy measure in English and Spanish. (DOCX) [file pone.0270103.s005.docx]

Coping Self-Efficacy Scale

When things aren’t going well for you, or when you’re having problems, how confident or certain are you that you can do the following:

| I am not at all certain I can do. |  |  |  | I am moderately certain I can do. | |  |  |  | I am certain I can do. |
| --- | --- | --- | --- | --- | --- | --- | --- | --- | --- |
| 1 | 2 | 3 | 4 | 5 | 6 | 7 | 8 | 9 | 10 |

**For each of the following items, write a number from 0 –10, using the scale above.**

**When things aren’t going well for you, how confident are you that you can:**

1. Keep from getting down in the dumps. ______
2. Talk positively to yourself. ______
3. Sort out what can be changed, and what cannot be changed. ______
4. Get emotional support from friends and family. ______
5. Find solutions to your most difficult problems. ______
6. Break an upsetting problem down into smaller parts. ______
7. Leave options open when things get stressful. ______
8. Make a plan of action and follow it when confronted with a problem. ______
9. Develop new hobbies or recreations. ______
10. Take your mind off unpleasant thoughts. ______
11. Look for something good in a negative situation. ______
12. Keep from feeling sad. ______
13. See things from the other person’s point of view during a heated argument. ______
14. Try other solutions to your problems if your first solutions don’t work. ______
15. Stop yourself from being upset by unpleasant thoughts. ______
16. Make new friends. ______
17. Get friends to help you with the things you need. ______
18. Do something positive for yourself when you are feeling discouraged. ______
19. Make unpleasant thoughts go away. ______
20. Think about one part of the problem at a time. ______
21. Visualize a pleasant activity or place. ______
22. Keep yourself from feeling lonely. ______
23. Pray or meditate. ______
24. Get emotional support from community organizations or resources. ______
25. Stand your ground and fight for what you want. ______
26. Resist the impulse to act hastily when under pressure. ______

Escala de Autosuficiencia al Enfrentar Problemas (Coping Self-Efficacy)

Cuando las cosas no van bien o cuando tiene problemas, ¿qué tan seguro o segura se siente de poder hacer lo siguiente?

| No me siento para nada seguro/a de poder hacerlo. |  |  |  | Me siento más o menos seguro/a de poder hacerlo. | |  |  |  | Me siento muy seguro/a de poder hacerlo. |
| --- | --- | --- | --- | --- | --- | --- | --- | --- | --- |
| 1 | 2 | 3 | 4 | 5 | 6 | 7 | 8 | 9 | 10 |

Por cada una de las afirmaciones, escriba un número del 0-10 usando la escala de arriba.

Cuando las cosas no van bien, que tan seguro/a se siente de poder:

1. Evitar que mi ánimo caiga por el suelo por un tiempo prolongado ______
2. Hablarse positivamente a usted mismo/a ______
3. Separar lo que se puede cambiar de lo que no se puede cambiar ______
4. Recibir apoyo emocional de sus amigos y familia ______
5. Encontrar soluciones a sus problemas más difíciles ______
6. Dividir un problema muy angustiante en partes más pequeñas ______
7. Dejar opciones abiertas cuando las cosas se ponen estresantes ______
8. Crear un plan de acción y seguirlo cuando se enfrenta con un problema ______
9. Desarrollar nuevos pasatiempos o actividades recreativas ______
10. Despejar la mente de pensamientos negativos ______
11. Buscar lo positivo en una situación negativa ______
12. Evitar sentirse triste ______
13. Ver las cosas desde el punto de vista de la otra persona durante una fuerte discusión ______
14. Tratar otras soluciones a sus problemas cuando las primeras soluciones no funcionan _____
15. Evitar sentirse molesto/a por pensamientos desagradables _____
16. Hacer nuevos amigos _____
17. Conseguir que sus amigos le ayuden con lo que necesita _____
18. Hacer algo positivo para usted mismo cuando se siente desanimado/a _____
19. Aclarar su mente de pensamientos desagradables _____
20. Pensar en una parte del problema a la vez _____
21. Visualizar una actividad o un lugar agradable _____
22. Evitar sentirse solo/a _____
23. Rezar o meditar _____
24. Recibir apoyo emocional de organizaciones o recursos comunitarios _____
25. Mantenerse firme y luchar por lo que quiere _____
26. No actuar impulsivamente cuando esta bajo presión _____
