# Supplementary material for: Does individual advocacy work?: A research and evaluation protocol for a youth anti-sex trafficking program
Source: PLoS One. 2022 Jun 29;17(6):e0270103. doi: 10.1371/journal.pone.0270103 (PMC9242468; doi:10.1371/journal.pone.0270103)
Supplement: Attachment — Submitted filename: Response to Reviewers.docx [file pone.0270103.s006.docx]

March 14, 2022

To the members of the *PLOS ONE* Editorial Board,

Thank you for your time and energy in reviewing our manuscript, titled *Does Individual Advocacy Work? A Research and Evaluation Protocol for a Youth Anti-Sex Trafficking Program.* We appreciate the comments and suggestions that were made to help us improve the manuscript, and we feel that the attached revision addresses each of the points raised by the reviewers. Below is a point-by-point summary of all the changes that we have made to the manuscript; there is also a marked-up copy of the manuscript (“Revised Manuscript with Track Changes”) and a clean copy included in this revision (“Manuscript”).

One thing we would like to note here is that when we first submitted the article for review, data collection was ongoing. At this point, data collection has finished, and we continue to conduct analyses on the data. We have updated all discussion to past tense to reflect the closure of the study period. We also removed one source of data collection – the client satisfaction survey – because the state of Texas discontinued this practice before data collection concluded.

*Editor Comments:*

1. To begin, the editors asked for an updated financial disclosure. Please include the following statement in the revised version of this manuscript: “This project was supported by a Victims of Crime Act (VOCA) federal pass-through grant awarded to the Office of the Texas Governor (OOG) Child Sex Trafficking Team and Traffick911. The sponsor of this protocol – Traffick911 – has assisted with the study design and data collection plan described in this protocol. Traffick911 administrators have approved of the protocol described herein. The original funders of this project, including VOCA and the OOG, had no role in study design, data collection and analysis, decision to publish, or preparation of the manuscript. The opinions, findings, conclusions, and recommendations expressed in this publication are those of the authors and do not necessarily reflect the views of VOCA, OOG, or Traffick911. ([www.traffick911.com](http://www.traffick911.com))”
2. The editors also asked that we update the file names to match the style requirements of PLOS ONE. We have ensured that each file name matches the standard requirement for file names. We have also placed all figures and supplements into individual files, per request.
3. The editors also asked us to check all of our files against the style guide. We have updated the files to match the templates requested by PLOS ONE (i.e. updating reference style, figure labeling, heading and subheading style, captions, supplemental file information, etc.).
4. Finally, the editors asked that our ethics statement only appear in the Methods section of the manuscript. We have moved the statement to the beginning of the Methods section. We retained the statement about IRB exemption within the Methods section, as this statement was not specifically related to Ethics but instead provided important information about the IRB.

*Reviewer One Comments:*

1. “On page 2, the statistic for 300,000 at-risk youth should be clarified, as the bounds by which youth are considered at-risk are not the same as those used later in the Methods section to determine which individuals are “at risk” and eligible for the program. The text should be revised in these introductory paragraphs and in the Methods to make it clear to the reader that this discrepancy exists.”

We addressed this comment by updating the 300,000 figure entirely, since the US government is now acknowledging that estimate data is flawed. We changed the reference to a reference from 2018 that acknowledges that there are likely thousands of victims based upon the same risk factors that we later use to determine risk (i.e. running away, homelessness, child abuse/neglect, child welfare and juvenile justice involvement).

2. Abstract and throughout the main text – “female youth” is used. Do the authors mean girls? Or will the program include all genders of individuals who were assigned female at birth (e.g. cisgender girls, transgender boys, non-binary youth, gender non-conforming youth, etc.)? This seems to be answered on page 8 when describing the inclusion criteria. However, there is a mismatch in the terms that should be corrected. “Female” youth should be changed to “cisgender girls” and “male” youth changed to “cisgender boys” throughout the text.”

Thank you for pointing out this error. We have updated the text throughout to reflect the reviewer’s suggestion.

3. “There is not a section for limitations of the protocol, the authors should make some mention (whether as part of the introductory paragraphs or in describing the inclusion criteria) of how the focus of this study on only cisgender girls may influence the scope of their findings and widen the systemic gaps already existing in the field studying minor sex trafficking/CSE. Though the authors make a reasonable case for this exclusion on page 8, it should be clear how this decision will impact and limit the scope and applications for their work.”

Noted – we did not add a limitations section to the protocol because we felt that a limitations section would be more appropriate for an article that presented results. However, we did add this statement to page 8, per the reviewer’s suggestion: “It should be noted, however, that future application of this protocol should make every effort to include cisgender boys and trans youth in data collection and analysis, as the exclusion of these subpopulations limits the scope of study findings and widens the systemic research gaps between cisgender girls, cisgender boys, and trans youth within the CSE field.” Throughout the article, per reviewer 3’s suggestions, we mentioned where some components of the protocol could further limit the eventual results of the study.

4. “Figure 2 and page 12 text: it is not wholly clear from the in-text explanation why the two conditions in Figure 2 (age and disability status) are the decision points for whether “the game” is used as part of the URICA form. As the authors have noted that the language may need to shift away from the “the game” for clients who would find it psychologically triggering, these conditions do not seem to capture all cases in which such language would be triggering or inappropriate. It would be helpful for the authors to explain the rationale for these conditions further.”

We had many conversations with the program staff about how to decide which tool to use; ultimately, it was the program staff who developed this decision tree in order to meet their programming and client needs. We had significant concerns that, in the absence of a decision tree, advocates would never ask clients about sex selling and trading behaviors at all, since we identified this as an issue within Traffick911 programming (an unwillingness to even bring up trafficking at all with clients, despite this being within the mission of the agency – out of a misunderstanding of trauma-informed care principles). Anyhow, this is what we landed on when working collaboratively with program staff. We have added this explanation to this section, and we’ve also added a sentence to urge future researchers to work collaboratively with partnering agencies to develop their own decision trees to match the program/clients.

5. “Some clarification is needed throughout the text around the program goals, outcomes measured, methods, etc. for individuals in each of the following subpopulations of the program: a) those who have experienced CSE previously but are not currently experiencing CSE, b) those who are currently experiencing CSE, and c) those who are “at risk” for CSE based on explained criteria. A large portion of the instruments and the outcomes (URICA especially) and the organizational materials (Figure 1) seem to assume that clients will all be currently engaged in CSE/”the game” and prioritize exiting exploitation. However, these assumptions do not fit or apply to those who have a history of CSE but are not currently experiencing it or those who are just “at risk” of CSE. Additionally, the abstract states that the population of interest is just those “at-risk for sex trafficking” which seems to contradict with the main text. Thus, throughout the text (abstract, main text, appendices) it would be helpful to have the authors clarify how the instruments will be applied to each sub-population and to note specifically how the central focus around “readiness to leave CSE” (stated in the abstract) applies.”

We have clarified within the abstract, main text, and appendices that the cisgender girls included in this study are a) recently-identified sex trafficking survivors, and b) at-risk for revictimization. We’ve made sure to clarify explanations of risk within the context that these girls are at-risk for returning to “the life”, so services and evaluations are geared towards assessing readiness to change and to exit. We also added a citation and explanation related to “very high risk” youth as identified by the CSE-IT, and how services are the same by subgroup because the subgroups are conceptualized by the agency as one and the same.

*Reviewer Two Comments:*

This reviewer had several specific comments related to the Spanish versions of the protocol’s surveys. While we will not reply to each point raised about the Spanish translations of the surveys because they were so specific, we very much appreciate the detailed suggestions and recommendations the reviewer made. We were surprised to learn there were so many errors, as we hired a professional translator to complete the translations for the project.

We reached out to Esperanza United (<https://esperanzaunited.org/en/>), a nonprofit that mobilizes Latinx communities to end gender-based violence, to request technical assistance with the translated surveys. We also provided the reviewer’s comments to the assigned translator. While the translator was not a CSE survivor, she was specifically trained to provide this type of technical assistance within the anti-trafficking field. She edited and validated the translations for us, to match both the suggestions of the reviewer and her own knowledge of GBV, trafficking, and the Latinx community. Importantly, we removed these translated surveys from the main document, since they are meant to be supplemental information. Each survey called out in the main document is now in its own supplemental file, so there are no tracked changes for these surveys to show exactly where the translations were fixed. However, we have gone through them to the best of our ability to ensure that the reviewer’s specific concerns were addressed in the new versions of these surveys, uploaded as supplemental files.

The reviewer also commented, “Most of the Spanish phrases sound strange with technical words that translated literally into Spanish are difficult to understand, do not sound natural, or may not even make sense. In addition to revising the Spanish and validating it with Spanish-speaking victims, it may be a good idea to add the recommendation or instructions to the questionnaire that the questionnaire is answered with the help of professionals to help ensure victims understand the questions correctly.”

We believe that the translation from Esperanza United will address the first portion of the reviewer’s comment above. Of note, the hired translator decided to retain “el juego” because of best practice guidelines offered by DHS (see [https://www.dhs.state.il.us/OneNetLibrary/27897/documents/Initiatives/HumanTrafficking/El-Trafico-de-Seres-Humanos-Glosario-de-Terminos.pdf](https://urldefense.com/v3/__https:/www.dhs.state.il.us/OneNetLibrary/27897/documents/Initiatives/HumanTrafficking/El-Trafico-de-Seres-Humanos-Glosario-de-Terminos.pdf__;!!K6Z8K8YTIA!SIiof4YQvE_ATBuves3LbdvlldF6wlOpB_gaH4iHJGLgsRx7c7NSUSW3Wlsv96Zj$)). We also added a sentence to the protocol to recommend that all Spanish-language surveys ought to be completed with the help of professionals to ensure survivors understand the questions correctly.

*Reviewer Three Comments:*

1. “The first paragraph of the Methods section discusses how the Texas Governor’s Child Sex Trafficking Team was focused on creating MDTs, yet there is no content on how Traffick911 relates to MDTs. Is Traffick911 an MDT? How does Traffick911 coordinate services across systems? Who are their partners when responding to reports of child sex trafficking? Since MDTs are referenced throughout the protocol, clarification is crucial.”

We have clarified in the Methods section that Traffick911 plays a central role on several different MDTs located in Traffick911’s four-county service area. In this same section, we’ve also added some detail and discussion of how Traffick911 coordinates services and partners within the MDTs.

2. “The logic model and associated descriptions are incredibly illustrative and helpful to readers. What is missing is a discussion about why Traffick911? What about the organization positions them to administer this individual advocacy program? I can think of many reasons, including central location, current service offerings, connections in community, capacity, etc. but having these explicitly outlined in the paper would be helpful.”

We added this explanation to the “study design” section of the protocol, which explains both why Traffick911 is positioned to offer these services, and why they were selected for the evaluation: “Traffick911 was selected for this outcome evaluation because it received grant funding from the CST to explore its services and to potentially develop a service and evaluation model for other grantees to follow; Traffick911 received this grant because of its capacity to manage an evaluation, its location within a highly-populated region of Texas, its history of providing CSE advocacy services, and its reputation with the state and area MDTs.”

3. “Additionally, information about Traffick911’s current capacity for client direct services and how it relates to the proposed enrollment numbers would be important to include. Is there any data on the short-term outcomes outlined in the logic model? While available evaluation data may be limited for a number of reasons, including such information in the paper would provide readers with greater context about the reach/effectiveness of existing services.”

While we agree that this information could provide more context for the protocol, we hesitate to include any results of the evaluation because results fall outside the scope of the protocol. We plan to publish complete results in a manuscript to be developed in late spring/early summer, as analysis is ongoing. However, in order to provide a small amount of context about the scope of the study, we included the following statement: “Although a presentation of evaluation results falls outside the scope of this protocol, early analyses show that of the 95 clients included in the evaluation, each client had received roughly 93 advocacy services during their time in the program.”

4. “It also seems some of the outcomes of the logic model are listed specifically to address the goals of the proposed advocacy program (e.g. readiness to change, self-efficacy, social support). Is the logic model presented for the individual advocacy program, or is the logic model adopted by Traffick911 as an organization? Initially, I thought it was the latter, as the goals of the program are at the individual level but would appreciate clarification on when this logic model was adopted and motivating factors.”

We have added several sentences to the discussion of the logic model. This discussion clarifies that the logic model is for the agency, whose sole offering is the advocacy program. We’ve also added information about why the logic model was adopted.

5. “Given the study is happening during the pandemic, are there any implications that need to be included in the protocol specific to this time period? Are there aspects of the protocol that would be unnecessary if not in a global pandemic?”

We added a statement about the implications of the pandemic on the protocol, immediately following the statement about the time frame for the study.

6. “Does parental consent pose any challenges when providing services to at-risk youth, particularly those who run away from their home? What implications does this have for the study?”

We added a statement about the possible issues stemming from parental consent.

7. “When describing development plans and other measurement tools, there is mention the form is available in both English and Spanish; however, diversity of language is not described under the Study Site section This info would be critical to assessing feasibility of implementing the program/ measuring outcomes using existing tools.”

We have added data related to language diversity – almost 40% of Dallas residents are native Spanish speakers. The numbers are similar for Tarrant County and Fort Worth. The vast majority of clients are from Dallas County for this protocol.

8. “How have the results from the URICA been reported in previous publications? Specifically, has the readiness equation been used before? The equation seems appropriate, but citations would strengthen this section.”

We included an explanation that the equation is the one developed by the URICA authors; we also added three citations to demonstrate that this equation is in use with other populations and targeted issues for behavior change (i.e. substance use, smoking cessation, parental behaviors).

9. “Traffick911’s theory of change is referenced in the Self-efficacy survey section; however, no citation/figure is included. The theory of change would be an excellent addition to the protocol, if available.”

The theory of change is presented within the logic model. There is not a separate write-up for the agency’s theory of change, but essentially, it is the IEP model that guides the logic model, and is represented within the outcomes and activities tracked. We’ve added a brief explanation to the discussion of the logic model to describe the connection.

10. “In Figure 2, how will Traffick911 staff know if a client has a cognitive disability? Is this information obtained from partner agencies?”

The agency does not have a uniform way to assess this or obtain the information. We have added this statement to caution researchers on the decision tree and its limitations, while encouraging researchers and practitioners to collaboratively create their own decision trees.

11. “The analyses section would benefit from additional information on if/how confounding factors may be controlled for during regression analyses.”

Since confounding factors can be controlled by multivariate analyses, we clarified that we will use multivariate linear, logistic, and Poisson regressions to control for confounding factors.

We are hopeful that the above-described revisions are satisfactory, but if not, please let us know and we will gladly make additional changes. We believe that this protocol has the potential to provide meaningful guidance to investigators and advocates who are working in anti-trafficking programs nationwide, and we look forward to hearing back from you about the manuscript and its potential for publication.

Sincerely,

Mary K. Twis, PhD, LMSW-AP

Assistant Professor

Department of Social Work

Texas Christian University
